# Midwives’ professional and personal experiences during the COVID-19 pandemic in Europe: The set-up of the ‘CoroVie’ project

**DOI:** 10.18332/ejm/122694

**Published:** 2020-06-19

**Authors:** Ans Luyben, Valerie Fleming, Joeri Vermeulen

**Affiliations:** 1 Centre for Midwifery, Maternal and Perinatal Health, Faculty of Health & Social Sciences, Bournemouth University, Bournemouth, United Kingdom; 2 Department of Health Services Research, University of Liverpool, Liverpool, United Kingdom; 3 School of Nursing and Allied Health, Liverpool John Moores University, Liverpool, United Kingdom; 4 Department Health Care, Knowledge Centre Brussels Integrated Care, Erasmus Brussels University of Applied Sciences and Arts, Brussels, Belgium; 5 Faculty of Medicine and Pharmacy, Department of Public Health, Biostatistics and Medical Informatics Research group, Vrije Universiteit Brussel, Brussels, Belgium

**Keywords:** COVID-19, pregnancy, childbirth, midwifery, midwives’ experiences, oral history project

**Dear Editor,**

At the end of December 2019, Chinese officials reported that an increasing number of people were being infected by a new virus (SARS-CoV-2, causing the disease COVID-19)^1^ and had become very ill and died. During the following months, this virus rapidly spread globally, and particularly affected people in Europe. By mid-March 2020 pandemic levels were reached, an extreme situation. The last pandemic to have such a devastating effect on Europe was the Spanish flu during 1918–1920.

Within a matter of weeks, the work of midwives changed dramatically^2^. Whereas it remained essential that they provided family-centred care, established good communication with mothers and offered emotional support and stress management, the environment in which midwives had to provide this care progressively changed in order to help prevent the spread of the virus^3^. In many European countries midwives called for personal protection and acceptable working conditions, so that they could keep the focus on safe and respectful care^4^. In some countries, midwives could not live with their families, while working in hospitals where patients with COVID-19 where cared for. These extreme professional and personal challenges, during the pandemic and under the associated preventive measures taken, coincide with the fact that 2020 is the International Year of the Nurse and the Midwife. While the COVID-19 pandemic in particular affects the healthcare workforce, being at the centre of care and treatment, little is known about their own experiences so far.

In order to capture the daily experiences of working during the pandemic, both professionally as well as personally, we developed the project ‘CoroVie’. We had the desire to follow and document the evolving COVID-19 situation in Europe and to explore professional situations that midwives encountered in different settings. Moreover, we wanted to gain an extensive insight into a midwife’s personal experiences in daily life, as a daughter, mother, sister, neighbour and as part of the community, during this period. While pursuing the principles of an oral history project^5^, we used for narrative purposes a private Facebook^®^ group as a digital media platform and its interactivity , so as to capture the life experiences of midwives and create a community^6^. In March 2020, we invited several European midwives through personal contact to join this group. They were informed about the purpose of the study and were asked if they wished to share their experiences in a private Facebook^®^ group, by writing about their life as often as possible, in a letter or diary format. Currently, midwives from nine European countries are participating from a diversity of midwifery practices and education and research settings. The midwives write in their own language, with a preference for English, German, Dutch and French. However, as Facebook translation enables sharing and interaction, other languages are also welcome. ‘CoroVie’ will run until the end of June 2020. The insights gained from this project may provide an excellent opportunity to revalue the midwifery profession and highlight its importance during this moving International Year of the Nurse and Midwife.
